# Assessing the factor structure of the Eating Attitude Test-26 among undergraduate students in Malaysia

**DOI:** 10.3389/fnut.2023.1212919

**Published:** 2023-11-16

**Authors:** Walton Wider, Jasmine Adela Mutang, Bee Seok Chua, Jiaming Lin, Assis Kamu, Nicholas Tze Ping Pang

**Affiliations:** ^1^Faculty of Business and Communications, INTI International University, Nilai, Negeri Sembilan, Malaysia; ^2^Faculty of Psychology and Education, University Malaysia Sabah, Kota Kinabalu, Malaysia; ^3^Faculty of Science and Natural Resources, University Malaysia Sabah, Kota Kinabalu, Malaysia; ^4^Faculty of Medicine and Health Sciences, University Malaysia Sabah, Kota Kinabalu, Malaysia

**Keywords:** confirmatory factor analysis, Eating Attitude Test, eating disorder, undergraduate students, obesity

## Abstract

The objective of this study was to assess the factor structure of the 26-item Eating Attitude Test (EAT-26) through confirmatory factor analysis (CFA) among 1,084 undergraduate students in Malaysia. The initial findings indicated a lack of support for the proposed three-factor structure. Model modifications were made due to the inadequate initial fit. The fit of the model was significantly improved by excluding items with factor loadings below 0.40 and integrating residual covariances. In conclusion, it is necessary to make contextual modifications to the EAT-26 in order to effectively utilize it among Malaysian undergraduates. This highlights the significance of cultural adaptations in psychological instruments.

## Introduction

Eating disorders among young adults have increased in recent years as a result of the ongoing pandemic (1). Eating disorders are defined as a persistent irregularity of eating or eating-related behaviors that leads to abnormal food consumption and severely harms physical and psychological health in the Diagnostic and Statistical Manual of Mental Disorders, 5th edition (DSM-5). University students who had previously relied on outside food may have developed eating disorders as a result of the reinforcement of several strict lockdowns. Furthermore, a lack of physical activity during the pandemic may have resulted in abnormal food consumption among university students (2). Other negative effects of the pandemic on young adults included disruption of routines and increased psychological distress, both of which had a negative impact on food consumption (3).

Globally, when young adults graduate from high school and begin university, many of them will be living away from home for the first time (4). As a result, university life is an important time for shaping their eating attitudes and behaviors, which may be linked to weight gain and poor health in their emerging adulthood (5). South-East Asia’s lower- and upper-middle-income countries have a higher prevalence of disordered eating than other regions of the world (6). Many issues concerning eating habits confront university students. According to a Malaysian study, students preferred to eat foods that were easy to consume and were less expensive than those that were healthy and nutritious (7). Furthermore, it was discovered that, while the majority of the students ate meals on a daily basis, they had a tendency to skip meals, particularly breakfast. Another local study revealed a link between disordered eating and negative body image in young adults (8). University students face various academic and social challenges. These factors can exacerbate pre-existing personal issues, such as negative body image perceptions, which frequently originate from childhood experiences. Perceptions during childhood and adolescence can be intensified by influences from peers, media, and familial expectations. Entering university can intensify existing insecurities due to additional stressors. It is crucial to recognize and confront the possible exacerbation of body image issues in university settings.

A separate locally conducted study found that adolescents who were dieting and suffering from Post-Traumatic Stress Disorder (PTSD) were more likely to develop eating disorders (9). According to the study’s findings, 13.9% of university students in the sample were at risk of developing an eating disorder. A more recent Malaysian study found that one in every five university students had an eating disorder (5). Female students exhibited higher rates of disordered eating than male students. The study revealed that 22.9% of female university students had disordered eating, compared to 13.3% of their male counterparts. Among male students, only depressive symptoms were a reliable predictor of disordered eating. In females, however, depressive symptoms were the most important factor, followed by body size satisfaction and appreciation. These findings provide preliminary evidence that eating disorders increase the risk of depression, possibly due to an inability to control eating habits such as binge eating and caloric restraint. This can lead to a failure to achieve a desired physique, which can lead to depression.

The Eating Attitudes Test (EAT) was initially developed as a 40-item questionnaire designed to serve as a global index for symptoms of anorexia nervosa (10). The updated version, known as the Eating Attitudes Test-26 (EAT-26), is a more concise self-report questionnaire consisting of 26 items. Created by Garner et al. (11), the EAT-26 is geared toward examining attitudes and behaviors related to disordered eating. It measures three factor scores: (a) dieting, which assesses avoidance of fattening foods and preoccupation with weight loss; (b) bulimia and food preoccupation, which gauges intrusive thoughts about food and symptoms of bulimia; and (c) oral control, which measures self-control around eating and perceptions of societal pressure to gain weight. Studies have shown that the 14 items removed from the original EAT-40 were redundant, as evidenced by a strong correlation between EAT-26 and EAT-40 scores (12). Additionally, the third factor (oral control) is inversely related to weight and bulimia, while the second factor (bulimia and food obsession) is positively related to bulimia symptoms and higher body weight—both of which have implications for outcomes (13). The EAT-26 has been extensively utilized both in clinical and non-clinical settings to identify individuals at risk for eating disorders (14). The EAT-26 has been used by psychologist and clinical practitioners worldwide (15). It is worth noting that the tool has been translated into multiple languages and adapted for various cultural contexts, including Italian (16), Lebanese (17), Brazilian (18), Chinese (19), and Japanese (20).

Eating Attitudes Test-26 has increasingly been the instrument of choice in epidemiological studies examining eating disorders. In a study by Khaled et al. (21) involving 2,692 young female university students from Qatar—a Muslim-majority country in the Middle East—the factor structure and measurement invariance of the EAT-26 were examined across language and BMI categories. While the EAT-26 demonstrated strong internal consistency overall, the study revealed issues with the scale’s measurement properties related to language and BMI. These limitations suggest that when using EAT-26, or its shorter versions, to assess disordered eating among adolescent Arabic-speaking females with varying body weights, caution should be exercised due to these significant measuring difficulties.

A cross-sectional survey conducted at a large North Carolina university targeted undergraduates aged 18–25 years. The majority of participants were White (22). To calculate their BMI, the participants self-reported their weight and height. In addition to demographic information, participants completed the eating attitudes (EAT 26) scale to determine their proclivity for dieting. Only 12% of the subjects had disturbed eating behavior, which was lower than previously reported. Nonetheless, the findings support the widely held belief that improper dieting and disordered eating habits are common among college students, particularly females. At the same time, 10% of college males reported having disordered eating attitudes, demonstrating that the problem is not limited to young female students. In a Malaysian study by Edman and Yates (23), the researchers examined the relationship between eating habits and BMI among ethnic Chinese and ethnic Malay college students. The study utilized the EAT-26 and included 187 Malay and 80 Chinese students as participants. The study found no gender-based differences in disordered eating attitudes within either ethnic group. However, Malay students exhibited higher EAT-26 scores compared to their Chinese counterparts. This contrasts with research commonly conducted in Western settings, where gender differences in attitudes toward disordered eating are often observed.

Inconsistent and ambiguous factor-analytic results have fueled debate over the EAT-26’s factorial validity, as well as its general structure and utility as a screening tool in non-clinical samples. The differences in eating habits and body-image standards between English-speaking and non-English-speaking countries have been attributed primarily to cultural differences (24). Maïano et al. (25) investigated the factor structure of the EAT-26 in one of the largest groups (*n* = 1,779) of ethnically diverse Europeans and Africans in a series of experiments. This study’s sample included adolescent males and girls in France, aged 11–18 years, who could communicate in French. Using exploratory structural equation modeling (ESEM) and confirmatory factor analysis (CFA), the authors replicated the best-fitting six-factor model with 18 items from the EAT-26. These included social pressure to gain weight, fear of becoming overweight, eating-related control, eating guilt, food obsession, vomiting-purging behavior, and food preoccupation. Another less studied but critical feature is the demonstration of measurement invariance or equivalence across significant subgroups, i.e., the same construct is measured in each subgroup. While a few studies have found measurement inconsistency for the EAT-26 in English across ethnic groups, this has rarely been investigated for translations (26). Before presenting evidence of cultural group differences and similarities, linguistic measurement invariance in the mother tongue should be demonstrated. Without it, it is impossible to rule out the possibility that observed group differences are not transferable across languages or cultures.

Eating Attitudes Test-26 factorial validity and utility as a screening tool for eating disorders in non-clinical samples have been called into question due to its inconsistent and ambiguous factor-analytic results. It has been hypothesized that cultural differences, such as varying eating habits and body image standards between English-speaking and non-English-speaking countries, may contribute to these inconsistencies. In light of these considerations, the aim of this study is to examine the factor structure of the EAT-26 in the context of undergraduate students in Malaysia.

## Methods

### Participants and procedure

The participants in this study consisted of 1,084 undergraduates from one of the public universities in Sabah who were recruited through the use of a convenience sample. Prior to data collection, the EAT-26 was translated into Malay language by the researchers, who then asked two psychologists who are fluent in both English and Bahasa Malaysia in translating the translated instrument (the Malaysian version) back into English using a back translation technique (27). Informed consent was obtained from each student, ensuring they understood the purpose of the study and their rights as participants. The data was collected from first to third-year students who took the faculty’s compulsory courses. The data collection was conducted in one of the lectures of their compulsory course and the questionnaires were distributed to the students by the researcher after the lecture ended.

### Measure

The Malay version of EAT-26 was used to validate its applicability for assessing disordered eating attitudes and behaviors among undergraduate students in Malaysia. The EAT-26 is a 26-item questionnaire that assesses three major areas of disordered eating attitudes and behaviors: dieting behavior (e.g., “I am preoccupied with a desire to be thinner”), bulimia and food preoccupation (e.g., “I give too much time and thought to food”), and oral control (e.g., “I find myself eating when I’m not hungry”). The items are scored on a 6-point Likert scale, with higher scores indicating more severe disordered eating attitudes and behaviors. The EAT-26 is a validated and reliable measure of disordered eating attitudes and behaviors. Cronbach’s alpha coefficients ranged from 0.77 to 0.91 across various populations, indicating good internal consistency (11, 28, 29). Over a 4-week period, the EAT-26 was also shown to have good test-retest reliability, with intraclass correlation coefficients ranging from 0.77 to 0.92 (11, 28). The EAT-26 has been used in a variety of settings to identify people at risk for eating disorders and to evaluate the efficacy of interventions. It has been demonstrated to be sensitive to changes in eating attitudes and behaviors following treatment (30) and to have good predictive validity for identifying people at risk for eating disorders (11, 28).

### Data analysis

Confirmatory factor analysis was used to test whether the suggested three-factor model of the EAT-26 could be confirmed among Malaysian undergraduate students, using the IBM SPSS AMOS 23 Program (maximum likelihood estimation method). To evaluate the model’s fit to the data, Hu and Bentler (31) suggested using Comparative Fit Index (CFI > 0.90), Tucker-Lewis Index (TLI > 0.90), Root Mean Square Error of Approximation (RMSEA 0.05–0.08), and Standardized Root Mean Square Residual (SRMR 0.08). Furthermore, the EAT-26’s reliability was assessed using the internal consistency Cronbach alpha method.

## Results

### Respondents’ demographic characteristics

The participants’ mean age was 21.7 years (SD = 1.00). The majority of participants (68.1%) were female, with 31.9% being male. In terms of ethnicity, the largest group was Bumiputera Sabah (51.6%), followed by Malays (20.8%), Chinese (11.2%), Indians (6.5%), Bumiputera Sarawak (7.5%), and others (2.6%). In terms of academic year, Year 3 (74.0%) had the most participants, followed by Year 2 (13.6%), Year 4 (7.2%), and Year 1 (5.4%). Table 1 summarizes the sample’s demographic characteristics.

**TABLE 1:** Demographic information about respondents (*N* = 1,084).

	Frequency	Percentage (%)/mean (SD)
Age		21.7 years (1.00)
**Gender**		
Male	346	31.9
Female	738	68.1
**Ethnicity**		
Malay	225	20.8
Chinese	121	11.2
Indian	70	6.5
Bumiputera Sabah	559	51.6
Bumiputera Sarawak	81	7.5
Others	28	2.6
**Academic year**		
Year 1	57	5.4
Year 2	147	13.6
Year 3	802	74.0
Year 4	78	7.2

## Confirmatory factor analysis

Confirmatory factor analysis with maximum likelihood estimation was used to evaluate the EAT-26 factor structure. However, the results revealed that the hypothesized factor structure did not match the current dataset well. The fit indices, in particular, did not meet the acceptable thresholds established by Hu and Bentler (31). The CFI was calculated to be 0.662, the TLI to be 0.629, the RMSEA to be 0.102, and the SRMR to be 0.087.

**FIGURE 1:**
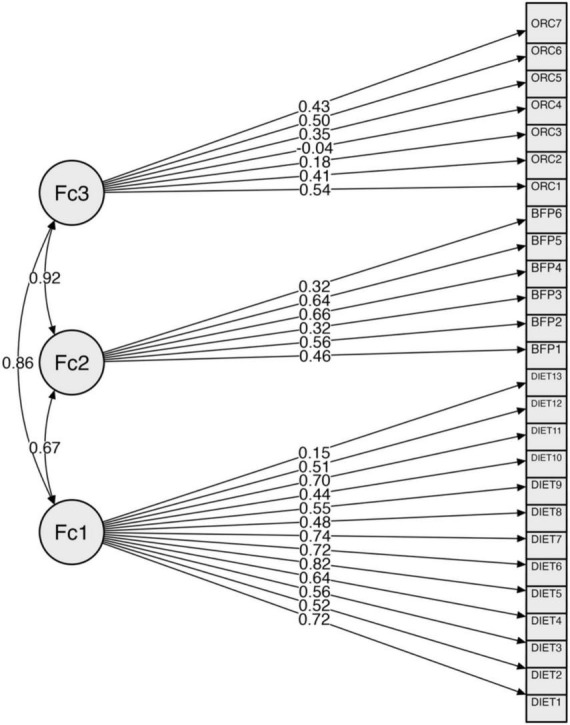
Factor structure with maximum likelihood estimation.

Despite the misalignment of the overall structure, a closer examination of individual items yielded more encouraging results. Except for the ORC4 item, all factor loadings ranged from 0.15 to 0.82 and were statistically significant (as shown in Table 2 and Figure 1). These findings indicate that the majority of the items were successfully loaded onto their designated factors. Furthermore, factor correlations were found to be statistically significant, indicating a moderate to high level of interdependence between the factors. Table 3 details these correlations. The significance of these correlations necessitates additional investigation to ensure that there is no undue overlap that could affect the discriminant validity of the factors.

**TABLE 2:** Standardized coefficients and associated data.

Factor	Indicator	Symbol	Estimate	SE	*z*-Value	*p*	95% Confidence interval	
							**LL**	**UL**
Factor 1	DIET1	λ11	1.129	0.043	26.092	<0.001	1.044	1.214
	DIET2	λ12	0.699	0.040	17.366	<0.001	0.620	0.778
	DIET3	λ13	0.653	0.034	19.208	<0.001	0.587	0.720
	DIET4	λ14	0.786	0.035	22.553	<0.001	0.718	0.855
	DIET5	λ15	1.338	0.043	31.380	<0.001	1.255	1.422
	DIET6	λ16	1.148	0.043	26.659	<0.001	1.064	1.233
	DIET7	λ17	1.182	0.043	27.464	<0.001	1.098	1.267
	DIET8	λ18	0.567	0.036	15.730	<0.001	0.497	0.638
	DIET9	λ19	0.614	0.033	18.433	<0.001	0.549	0.679
	DIET10	λ110	0.525	0.036	14.614	<0.001	0.454	0.595
	DIET11	λ111	0.836	0.033	25.235	<0.001	0.771	0.901
	DIET12	λ112	0.604	0.035	17.228	<0.001	0.535	0.672
	DIET13	λ113	0.231	0.048	4.803	<0.001	0.137	0.325
Factor 2	BFP1	λ21	0.616	0.045	13.576	<0.001	0.527	0.705
	BFP2	λ22	0.669	0.039	17.170	<0.001	0.592	0.745
	BFP3	λ23	0.217	0.023	9.288	<0.001	0.171	0.263
	BFP4	λ24	0.779	0.036	21.523	<0.001	0.708	0.850
	BFP5	λ25	0.781	0.038	20.758	<0.001	0.707	0.854
	BFP6	λ26	0.265	0.028	9.426	<0.001	0.210	0.320
Factor 3	ORC1	λ31	0.545	0.032	17.228	<0.001	0.483	0.607
	ORC2	λ32	0.521	0.042	12.457	<0.001	0.439	0.604
	ORC3	λ33	0.266	0.053	5.039	<0.001	0.163	0.370
	ORC4	λ34	−0.063	0.060	−1.042	0.298	−0.180	0.055
	ORC5	λ35	0.487	0.046	10.524	<0.001	0.396	0.578
	ORC6	λ36	0.582	0.037	15.942	<0.001	0.511	0.654
	ORC7	λ37	0.507	0.039	12.929	<0.001	0.430	0.584

**TABLE 3:** Correlation between factors.

	Estimate	SE	*z*-Value	*p*	95% Confidence interval	
					**Lower**	**Upper**
Factor 1 ↔ factor 2	0.666	0.026	26.088	<0.001	0.616	0.716
Factor 1 ↔ factor 3	0.863	0.031	27.525	<0.001	0.802	0.925
Factor 2 ↔ factor 3	0.916	0.030	30.178	<0.001	0.856	0.975

Cronbach’s alpha was used to assess internal consistency for each of the factors. With a value of 0.867, factor 1 demonstrated commendable internal consistency. With a value of 0.670, factor 2 demonstrated acceptable consistency. Factor 3’s consistency, on the other hand, was relatively low and questionable, with a value of 0.587. The lower consistency score for factor 3 suggests that the items in this factor may need to be refined or reconsidered in order to improve their reliability.

### Model plot

**FIGURE 2:**
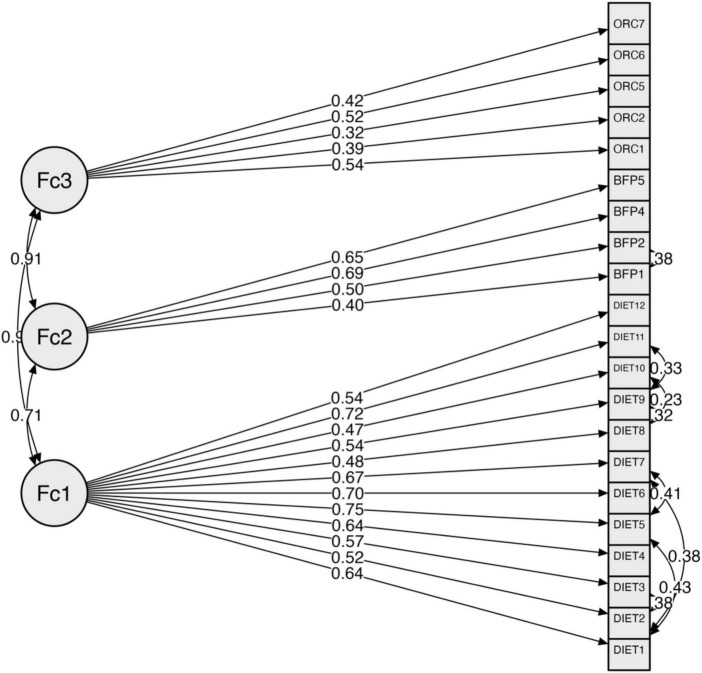
Factor structure with maximum likelihood estimation (after modification).

Modifications were deemed necessary due to the poor model fit. As shown in Figure 2 and Table 4, two major adjustments were made. To begin, items with factor loadings less than 0.40 were removed to improve model fit. Second, residual covariances were added based on modification indices. Following these changes, the model’s fit improved significantly: CFI = 0.870, TLI = 0.847, RMSEA = 0.075, and SRMR = 0.059. Internal consistency of the revised model improved significantly for factors 1 and 2. Factor 1’s consistency increased to 0.883 and factor 2’s to 0.701. However, factor 3’s consistency dropped slightly to 0.553, indicating that it should be scrutinized further or modified in future iterations.

**TABLE 4:** Correlation between factors (after modifications).

	Estimate	SE	*z*-Value	*p*	95% Confidence interval	
					**Lower**	**Upper**
Factor 1 ↔ factor 2	0.709	0.027	26.356	<0.001	0.656	0.762
Factor 1 ↔ factor 3	0.937	0.025	37.672	<0.001	0.888	0.986
Factor 2 ↔ factor 3	0.908	0.033	27.775	<0.001	0.844	0.972

## Discussion

The findings of this study suggest that the hypothesized three-factor model of the EAT-26 did not achieve an acceptable fit with the data from Malaysian undergraduate students. The fit indices (CFI = 0.662, TLI = 0.629, RMSEA = 0.102, SRMR = 0.087) fell short of the recommended criteria for an acceptable fit, as outlined by Hu and Bentler (31). This outcome aligns with previous research that has questioned the validity of the EAT-26 in various non-Western cultures, including those in Iran, Pakistan, Hong Kong, Israel, and the Arab world (21, 32–35). Notably, five items (ORC3, ORC4, BFP3, BFP6, and DIET13) on the EAT-26 had low factorial loadings (<0.40). This observation is consistent with the findings of Ocker et al. (36), who also reported poor fits for the three-factor models of the EAT-26. The inadequate fit may be attributable to a variety of factors, including cultural nuances, the use of non-clinical samples, and inherent limitations of the instrument itself. As a result, items with low factorial loadings may need to be revised or excluded from the questionnaire. Further corroborating the concerns about the EAT-26, multiple studies have identified poor internal consistency for the scale [e.g., (36, 37)]. Taken together, these findings cast doubt on the reliability and validity of the EAT-26 as a measure of disordered eating attitudes and behaviors among Malaysian undergraduate students.

Eating disorders are a worldwide problem that can have a significant impact on an individual’s overall health and quality of life including university students. Several studies conducted on the incidence of eating disorders among Malaysian university students have revealed that a range of 11–43% of the student population is susceptible to developing an eating disorder (9, 38–40). The EAT-26 is a self-report measure that has been extensively researched to assess symptoms and concerns that are typical of eating disorders, and has frequently been employed as a screening instrument for evaluating the likelihood of developing an eating disorder. The EAT-26 assessment tool has the potential to be utilized in both clinical and non-clinical environments, without a specific emphasis on eating disorders.

In light of the results we’ve discussed earlier, it is crucial to consider the broader context of eating disorders, notably among university students. The EAT-26 assessment tool, which we utilized in our study, is designed to screen for symptoms and concerns typical of eating disorders. It is a versatile tool applicable in both clinical and non-clinical settings.

As detailed in our results section, the EAT-26 assesses three key factors: dieting, bulimia, and oral control. Although the tool was originally validated in Western contexts, it has been adapted for various cultures (41–43). Its psychometric properties have been both lauded and criticized in academic literature. While some studies affirm its robustness (36, 41, 43, 44), others question its reliability and validity (21, 32, 36, 42). One area that remains ambiguous is the cultural applicability of the EAT-26 in Malaysia, especially among university students—a primary focus of our study. Although we did not employ qualitative methodologies in this research, future studies could consider using “Think-aloud” techniques to probe deeper into the language and cultural nuances affecting the EAT-26’s reliability and validity in non-Western contexts (45).

Our findings suggest that the cultural milieu in Malaysia might significantly impact the EAT-26’s validity. The cultural norms around food and hospitality in Malaysia, as highlighted by the concept of “Third Space” (46) and work by Perry (47), could render some items in the Oral Control factor incongruent with local customs. Phrases like “please eat more and enjoy the food” are indicative of these deeply rooted cultural norms. Another aspect to consider, as revealed in our analysis, is the challenge of linguistic translation. The EAT-26 underwent adaptation from its original English version to the Malay language via back to back translation. However, this translation process might not have adequately captured the subtleties of local cultural nuances and lack of comprehensive cross-cultural adaptation process. In this approach, the questionnaire was translated from its original English version into, potentially resulting in certain questionnaire items becoming less relevant or difficult to interpret within the Malaysian culture context. Consequently, this limitation could pose significant concerns regarding the validity of the tool within this cultural setting, potentially being the primary factor hindering the interpretation of the CFA results. The absence of a more thorough cross-cultural adaptation process could compromise the questionnaire’s reliability and validity in the Malaysian context. This concern was supported by our results, which found poor factor loadings for items in the Bulimia and Food Preoccupation factors, likely due to our non-clinical sample. This aligns with recent studies that question the EAT-26’s applicability in non-clinical settings (48, 49). Moreover, critics argue that the EAT-26 may be overly complex for use in general health surveys (50).

Finally, the limitations of our study deserve acknowledgment. The cross-sectional design and convenience sampling method limit the findings’ generalizability. Additionally, the original validation sample for the EAT-26 differed significantly from ours, being a clinical female sample diagnosed with anorexia nervosa. Furthermore, the self-reported nature of our data introduces the potential for social desirability and recall biases, which could either underreport or exaggerate the psychopathology associated with eating disorders.

## Conclusion

The implications of this research warrant careful consideration, particularly in the context of shifting lifestyle and dietary patterns influenced by rapid urbanization and economic growth (51). Recent observations point to increasing rates of obesity (52), growing preferences for fast food and processed meals, more sedentary lifestyles, and heightened exposure to Western beauty standards that glorify thinness. Implementing early detection of eating disorders could serve as a cost-effective public health strategy, notably in educational environments where interventions are both practical and economically viable (53).

Our study contributes to a better understanding of the applicability of the EAT-26 questionnaire within a Malaysian context, which is crucial for the development of culturally relevant interventions and policies for treating eating disorders in this particular demographic. However, it is important to note that while the EAT-26 is a widely used tool for assessing abnormal eating behaviors, its factorial structure appears to be less robust, especially among the non-clinical sample of university students that we examined.

The homogeneity of our sample—comprised solely of university students, largely of the same age and predominantly single—poses limitations on the generalizability of our findings to the broader population. Additionally, our results raise questions about the potential influence of cultural factors on how individuals interpret the EAT-26 items. Further research is warranted to validate these preliminary observations and to assess the utility of the original three-factor structure of the questionnaire in various cultural contexts within Malaysia.

## Data availability statement

The raw data supporting the conclusions of this article will be made available by the authors, without undue reservation.

## Ethics statement

Ethical approval was not required for the studies involving humans because the study was conducted according to the guidelines of the Declaration of Helsinki and following academic ethics. The studies were conducted in accordance with the local legislation and institutional requirements. The participants provided their written informed consent to participate in this study.

## Author contributions

All authors listed have made a substantial, direct, and intellectual contribution to the work and approved it for publication.
